# Perinatal mortality associated with induction of labour versus expectant management in nulliparous women aged 35 years or over: An English national cohort study

**DOI:** 10.1371/journal.pmed.1002425

**Published:** 2017-11-14

**Authors:** Hannah E. Knight, David A. Cromwell, Ipek Gurol-Urganci, Katie Harron, Jan H. van der Meulen, Gordon C. S. Smith

**Affiliations:** 1 Department of Health Services Research and Policy, London School of Hygiene & Tropical Medicine, London, United Kingdom; 2 Royal College of Obstetricians and Gynaecologists, London, United Kingdom; 3 Department of Obstetrics and Gynaecology, University of Cambridge, NIHR Cambridge Comprehensive Biomedical Research Centre, Cambridge, United Kingdom; University of Manchester, UNITED KINGDOM

## Abstract

**Background:**

A recent randomised controlled trial (RCT) demonstrated that induction of labour at 39 weeks of gestational age has no short-term adverse effect on the mother or infant among nulliparous women aged ≥35 years. However, the trial was underpowered to address the effect of routine induction of labour on the risk of perinatal death. We aimed to determine the association between induction of labour at ≥39 weeks and the risk of perinatal mortality among nulliparous women aged ≥35 years.

**Methods and findings:**

We used English Hospital Episode Statistics (HES) data collected between April 2009 and March 2014 to compare perinatal mortality between induction of labour at 39, 40, and 41 weeks of gestation and expectant management (continuation of pregnancy to either spontaneous labour, induction of labour, or caesarean section at a later gestation). Analysis was by multivariable Poisson regression with adjustment for maternal characteristics and pregnancy-related conditions. Among the cohort of 77,327 nulliparous women aged 35 to 50 years delivering a singleton infant, 33.1% had labour induced: these women tended to be older and more likely to have medical complications of pregnancy, and the infants were more likely to be small for gestational age.

Induction of labour at 40 weeks (compared with expectant management) was associated with a lower risk of in-hospital perinatal death (0.08% versus 0.26%; adjusted risk ratio [adjRR] 0.33; 95% CI 0.13–0.80, *P* = 0.015) and meconium aspiration syndrome (0.44% versus 0.86%; adjRR 0.52; 95% CI 0.35–0.78, *P* = 0.002). Induction at 40 weeks was also associated with a slightly increased risk of instrumental vaginal delivery (adjRR 1.06; 95% CI 1.01–1.11, *P* = 0.020) and emergency caesarean section (adjRR 1.05; 95% CI 1.01–1.09, *P* = 0.019). The number needed to treat (NNT) analysis indicated that 562 (95% CI 366–1,210) inductions of labour at 40 weeks would be required to prevent 1 perinatal death. Limitations of the study include the reliance on observational data in which gestational age is recorded in weeks rather than days. There is also the potential for unmeasured confounders and under-recording of induction of labour or perinatal death in the dataset.

**Conclusions:**

Bringing forward the routine offer of induction of labour from the current recommendation of 41–42 weeks to 40 weeks of gestation in nulliparous women aged ≥35 years may reduce overall rates of perinatal death.

## Abstract 2

In a cohort study using English Hospital Episode Statistics (HES) data, Hannah E Knight and colleagues examine perinatal mortality and other birth outcomes in association with labour induction at 39, 40, and 41 weeks.

## Author summary

**Why was this study done?:**

- National guidelines recommend that induction of labour is carried out between 41 and 42 weeks of gestation to prevent the risks associated with prolonged pregnancy. However, women having their first baby at age 35 years or over are at increased risk of pregnancy complications, including perinatal death.
- A recent randomised controlled trial demonstrated that induction of labour at 39 weeks of gestation has no short-term adverse effect on the mother or infant among nulliparous women aged 35 years or older. However, the trial was underpowered to address the effect of routine induction of labour on the risk of perinatal death.
- The present study aims to answer the question ‘Does routine induction of labour at or after 39 weeks of gestation reduce the risk of perinatal mortality in first-time mothers aged 35 years or older, compared with expectant management?’

**What did the researchers do and find?:**

- In this national cohort study of 77,327 first-time mothers aged 35 or older, induction of labour at 40 weeks of gestation was associated with a 66% lower risk of perinatal death (0.08% versus 0.26%) than expectant management.
- Perinatal death is a rare outcome even in this group and 562 inductions of labour at 40 weeks would be required to prevent 1 perinatal death.

**What do these findings mean?:**

- Bringing forward the routine offer of induction of labour from the current recommendation of 41–42 weeks to 40 weeks of gestation in this group of women may reduce overall rates of perinatal death.

## Introduction

Across industrialised nations, the proportion of births to women aged ≥35 years is rising [1,2]. In England and Wales, births to women aged ≥35 years have increased from 6% of all births in 1975 to 21% in 2015 [3]. There has also been an increase in the number of babies born to first-time mothers aged ≥35 years, which in 2015 accounted for 14% of all first-time births and 5.4% of all births in England and Wales [4].

Older women are at increased risk of pregnancy complications, including gestational diabetes, placenta praevia, and postpartum haemorrhage [5,6], and experience higher rates of intervention during labour and delivery [5,7]. After controlling for comorbidities, the risk of antepartum stillbirth at term is higher among women aged ≥35 years than among younger women [8] and is higher still for nulliparous women aged ≥35 years [9]. Observational data indicate that induction of labour at or before term may be beneficial because the risk of perinatal death is at its lowest for births between 38 and 39 weeks of gestation [9]. However, current United Kingdom guidelines recommend that induction for prolonged gestation is offered to women between 41 to 42 weeks of gestation, when the risk of stillbirth is 2 to 3 per 1,000 deliveries [10].

A recent randomised controlled trial (RCT) has shown that among nulliparous women aged ≥35 years, elective induction of labour at 39 weeks of gestation had no significant effect on the rate of caesarean section and no adverse short-term effects on maternal or neonatal outcomes compared with expectant management [11], but the trial was underpowered to examine the effect of induction of labour on the risk of perinatal death. Well-conducted observational studies have found that induction of labour at term is associated with decreased perinatal mortality in the general pregnant population [12]; however, none has been sufficiently powered to examine the impact on this specific age group known to be at increased risk. We employed a large English routine dataset to determine the association between induction of labour at ≥39 weeks and the risk of perinatal death among nulliparous women aged ≥35 years.

## Methods

We designed our methods to test the hypothesis that induction of labour at 39, 40, and 41 weeks reduced the risk of perinatal mortality among nulliparous women aged ≥35 years compared with expectant management (continuation of pregnancy to either spontaneous labour, induction of labour, or caesarean section at a later gestation).

### Details of ethics approval

The study is exempt from UK National Research Ethics Service (NRES) approval because it involved the analysis of an existing dataset of anonymised data for service evaluation. Hospital Episode Statistics (HES) data were made available by NHS Digital (Copyright 2015, re-used with the permission of NHS Digital. All rights reserved.) Approvals for the use of anonymised HES data were obtained as part of the standard NHS Digital data access process.

### Data source

We used the HES database to identify births in English National Health Service (NHS) hospitals. The HES database contains patient demographics, diagnostic and procedure information, and administrative data for each inpatient episode of care since 1997 [13]. A unique identifier enables studies to combine episodes of care that belong to the same patient.

In the HES database, for episodes related to childbirth, supplementary fields (the ‘maternity tail’) capture details including parity, birthweight, gestational age, onset of delivery, method of delivery, and birth outcome. Mothers’ delivery episodes were defined as records containing information about the mode of delivery in either the OPCS4 codes (R17–R25) or the maternity tail.

Diagnostic information is coded using the *International Classification of Diseases*, *10th Revision* (ICD10) [14], and operative procedures are coded using the UK *Office for Population Censuses and Surveys Classification*, *4th Revision* (OPCS4) [15]. The level of data completeness has improved over time [16,17] but varies across NHS hospitals: in 2014, data on onset of labour and gestational age were available in 85% and 82% of all delivery episodes, respectively [18].

### Study population

We included all nulliparous women aged 35 to 50 years delivering at 39 weeks of gestation or beyond, between April 2009 and March 2014. We excluded multiple births; women with preexisting comorbidities (hypertension, diabetes, and cardiac or lung disease); births complicated by fetal malpresentation, abdominal pregnancy, and placenta praevia; and pregnancies resulting in perinatal deaths due to congenital abnormality. We excluded records that were missing birth status, delivery onset, or gestational age. We also performed data quality assessments at the individual hospital level and excluded hospitals with suspected poor-quality data for these key data items (S1 Appendix, Text A). The characteristics of women excluded on the basis of these assessments were compared with those of the study cohort. Limiting birth cohorts in this way to include only hospitals with high completeness of recording has been demonstrated to be a valid way of constructing cohorts from routine hospital data [16,17]. The mothers’ delivery records were linked to the hospital records of their babies using probabilistic linkage [19] to obtain data on perinatal outcomes using the babies' birth records (e.g., for in-hospital perinatal death) and any subsequent hospital inpatient or A&E records (e.g., emergency neonatal readmission within 28 days). Induction of labour was defined as either surgical induction by amniotomy; medical induction, including the administration of agents either orally, intravenously, or intravaginally; or a combination of surgical and medical induction.

### Definitions of the induction and expectant management groups

For an observational study to appropriately examine the outcomes of induction of labour at different gestational ages, it is important to compare outcomes of women who have an induction of labour at a particular week of gestation (week *n*) with women who are expectantly managed, i.e., go on to deliver at a later gestation by any mode of onset, and not with women who labour spontaneously at the same gestation. There are 2 possible ways to define the expectant management group using observational data in which gestational age is recorded in weeks (see Stock et al. [12] for discussion):

1. women delivering at weeks >*n*
2. women delivering at weeks ≥*n*

For the primary analysis, we adopted the first approach, including women delivering at weeks >*n* following spontaneous or induced labour or prelabour caesarean. The robustness of this approach was then tested using a secondary analysis that used the alternative definition.

For each week of gestation examined, we excluded women if their labour was induced following an antepartum intrauterine death or prelabour rupture of membranes because, in both conditions, if labour does not begin spontaneously within 24 hours, the standard management is induction of labour [10]. However, we did not exclude women with these complications from the expectant management group if the event occurred after the week of gestation of induction in the induction group (at weeks >*n*). This followed a similar approach used in previous studies [12] and is supported by evidence that delays in the delivery of antepartum stillbirths or PROM are uncommon in UK hospitals [20]. Intrapartum stillbirths in all weeks ≥*n* were included in both groups.

### Outcomes

We examined the following neonatal outcomes: stillbirth, in-hospital perinatal death, birth injury, shoulder dystocia, hypoxia in labour, meconium aspiration syndrome, neonatal seizures, and emergency readmission to hospital within 28 days of birth. In-hospital perinatal death was defined as stillbirth or in-hospital neonatal death within 7 days of birth. We also recorded the following maternal outcomes: emergency caesarean section, instrumental delivery, third or fourth degree perineal tear, and emergency readmission to hospital within 28 days of delivery. To calculate readmission rates, births that occurred in the last 28 days of the study period were excluded. Details of the definitions used are given in S1 Appendix, Table A.

### Statistical analysis

All analyses were prespecified as described in the Methods section, with the exceptions of the exclusion of women with preexisting comorbidities and the use of Poisson rather than logistic regression. These modifications were made prior to the commencement of any statistical analyses; the rationale for each change is provided in S1 Appendix, Text B. We did not publish or pre-register a plan for this analysis.

We used proportions to summarise the distribution of pregnancy characteristics of induced and non-induced women and the chi-squared test for comparisons of variables between the groups. For each week of gestation, univariable and multivariable Poisson regression with robust standard errors was used to estimate the crude and adjusted effects of induction of labour compared with expectant management on each maternal and perinatal outcome. We chose not to use logistic regression because odds ratios overestimate the risk ratio for common outcomes [21]. The confounding variables included in all models were maternal age (years); ethnicity (white, Asian, black, other, or unknown); year of birth; baby’s sex (male or female); birthweight percentile according to UK 1990 fetal growth charts (<10th, 10th–90th, or >90th) [22]; and socioeconomic quintile according to the a Index of Multiple Deprivation (IMD) score, a measure that combines economic, social, and housing indicators [23]. The year of the birth was recorded as a linear variable to take into account changes in clinical practice over time. Estimates were adjusted for pregnancy-related conditions (pregnancy-induced hypertension, preeclampsia, or oedema; gestational diabetes; and fluid abnormalities) when these were found to have significant coefficients. No formal tests of interaction were done, and no adjustments were made for multiple comparisons. For both our primary and secondary analyses, we estimated the number of inductions of labour needed to prevent 1 perinatal death: number needed to treat (NNT) = 1/([induction of labour event risk]−[expectant management event risk]). All statistical tests were 2-sided, and the level of significance was set at *P* < 0.05. All analyses were performed in STATA version 14.1 (StataCorp, College Station, TX, United States).

## Results

**Fig 1:**
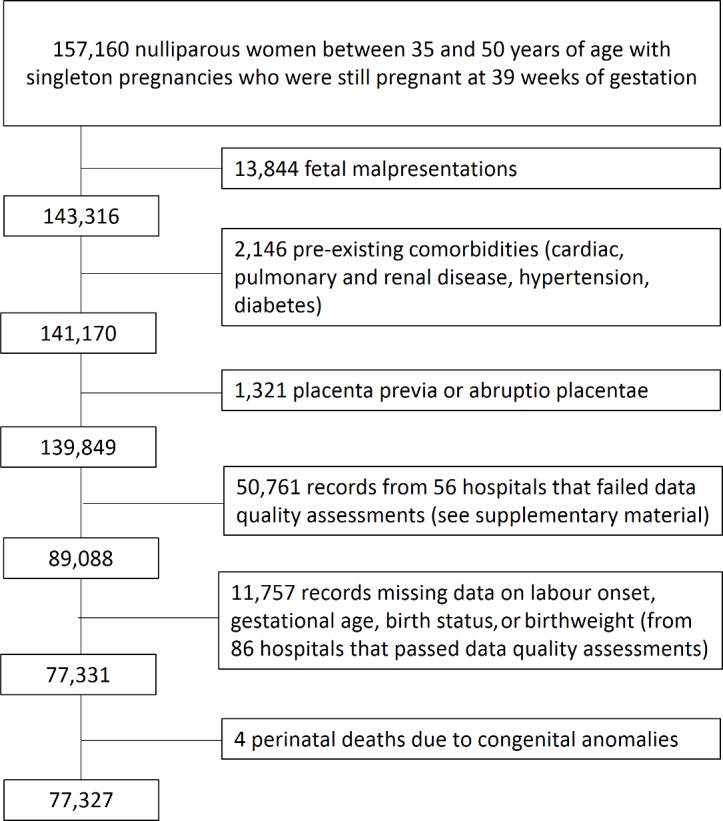
Cohort selection flow chart.

There were 77,327 women aged 35–50 years who met the inclusion criteria and gave birth in hospitals that passed the data quality assessments for key data items (Fig 1). Of these women, 25,583 (33.1%) were induced and 51,744 (66.9%) were not. Induction of labour rates among this group of women increased each year during the time period from 30.2% in 2009–2010 to 35.7% in the 2013–2014 cohort. Medical induction of labour was the principal method of induction throughout the time period (57.7% of inductions), with surgical and combined methods used less frequently (19.7% and 19.4% of inductions, respectively).

There were 50,761 eligible women who gave birth in hospitals that failed the data quality assessments for key data items required in the analysis, and these women were therefore not included in the study. The women included in the study shared similar characteristics to the excluded women who gave birth in hospitals with poor data quality (S1 Appendix, Table B). Hospitals that failed the data quality assessments were missing data on gestational age, birth status, and onset of labour in 40%, 25%, and 24% of records, respectively, compared with 11%, 10%, and 11% of records in hospitals that passed these assessments.

**Fig 2:**
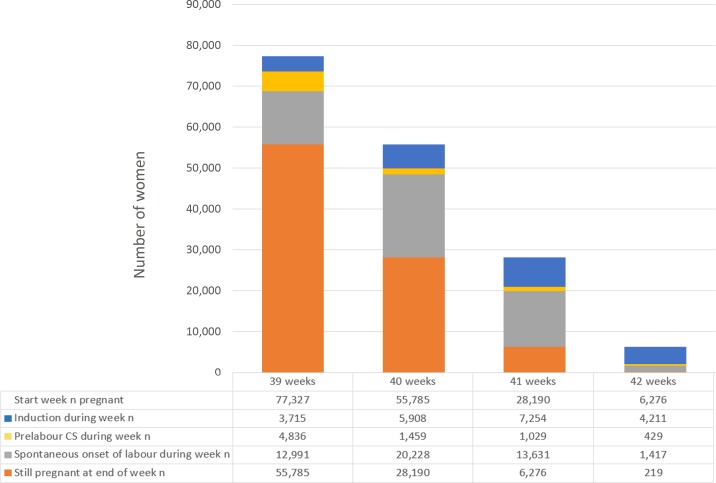
Composition of cohort by week of gestation. For each week of gestation (column), the primary analysis compares women who were induced at week *n* (blue column segment) with women who were expectantly managed, defined as those who delivered at weeks >*n* (orange column segment). The secondary analysis compares women who were induced at week *n* (blue column segment) with those who were expectantly managed according to the alternative definition, i.e., delivered at weeks ≥*n* (orange, grey, and yellow column segments). Abbreviation: CS, caesarean section.

Women who had labour induced were more likely to be over 40 years old, of white ethnicity, and to deliver infants in less than the 10th centile for birthweight (Table 1). They were also more likely to have acquired complications of pregnancy. Fig 2 describes the composition of the cohorts used for the primary and secondary analyses.

**Table 1:** Pregnancy characteristics of 77,327 women according to induction of labour.

Characteristic	Group	Not induced (*n* = 51,744) *n* (%)	Induced (*n* = 25,583) *n* (%)	Total *n* (%)
**Maternal age (years)**	35–39	43,691 (84.4)	19,880 (77.7)	63,571 (82.2)
	40–50	8,053 (15.5)	5,703 (22.3)	13,756 (17.8)
Maternal ethnicity*	White	37,507 (80.1)	19,341 (82.7)	56,848 (81.0)
	Asian	3,352 (7.2)	1,453 (6.2)	4,805 (6.8)
	Black	2,655 (5.7)	1,224 (5.2)	3,879 (5.5)
	Other	3,319 (7.1)	1,357 (5.8)	4,676 (6.7)
**Maternal SES quintile**	SES 1 (least deprived)	11,350 (21.9)	5,565 (21.8)	16,915 (21.9)
	SES 2	10,861 (21.0)	5,444 (21.3)	16,305 (21.1)
	SES 3	10,470 (20.2)	5,190 (20.3)	15,660 (20.3)
	SES 4	11,225 (21.7)	5,492 (21.5)	16,717 (21.6)
	SES 5 (most deprived)	7,836 (15.1)	3,890 (15.2)	11,726 (15.2)
**Year of birth**	2009	11,897 (23.0)	5,147 (20.1)	17,044 (22.0)
	2010	10,732 (20.7)	5,001 (19.6)	15,733 (20.4)
	2011	10,756 (20.8)	5,348 (20.9)	16,104 (20.8)
	2012	9,545 (18.5)	5,193 (20.3)	14,738 (19.1)
	2013	8,814 (17.0)	4,894 (19.1)	13,708 (17.8)
**Birthweight centile**	10–90th	43,538 (84.1)	21,164 (82.7)	64,702 (83.7)
	<10th	4,545 (8.8)	2,597 (10.2)	7,142 (9.2)
	>90th	3,661 (7.1)	1,822 (7.1)	5,483 (7.1)
**Sex of baby**	Male	21,311 (50.9)	13,245 (51.8)	39,556 (51.2)
**Pregnancy complications**	Preeclampsia	2,493 (4.8)	3,319 (13.0)	5,812 (7.5)
	Gestational diabetes	875 (1.7)	1,440 (5.6)	2,315 (3.0)
	Abnormal fluid volume	240 (0.5)	417 (1.6)	657 (0.9)

* Ethnicity was unknown in 7,119 (9.2%) of records. A missing category was included in the regression models.

Abbreviations: SES, socioeconomic status.

### Perinatal mortality

**Fig 3:**
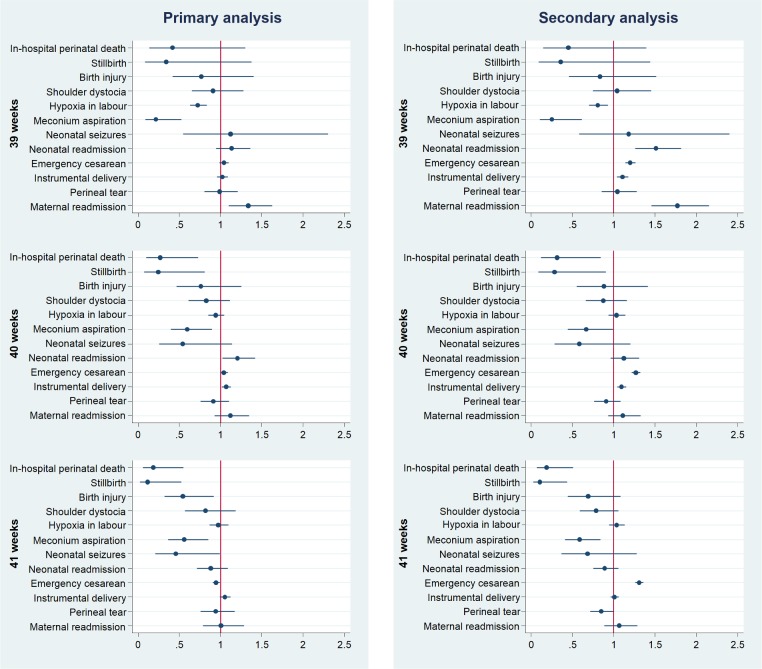
Perinatal outcomes after induction of labour compared with expectant management by week of gestation of induction of labour. Outcomes have been adjusted for potential confounders. Full details and results of all models are presented in Table 2 and S1 Appendix, Table C. The horizontal axis represents adjusted relative risk, with a relative risk <1 favouring induction of labour.

Labour induction from 40 weeks onwards was associated with a significantly reduced rate of both in-hospital perinatal death and stillbirth when compared with expectant management (Fig 3).

In the primary analysis, the adjusted risk ratio (adjRR) for in-hospital perinatal death associated with induction compared with expectant management was 0.33 (95% CI 0.13–0.80) at 40 weeks and 0.24 (95% CI 0.09–0.65) at 41 weeks (Table 2). However, there was no difference in the estimated adjRR for in-hospital perinatal death associated with induction at 39 weeks (0.37; 95% CI 0.12–1.15). Similar magnitudes of effect were observed for stillbirth, with an adjRR of 0.25 (95% CI 0.09–0.79) at 40 weeks and 0.18 (95% CI 0.05–0.65) at 41 weeks. The results of the secondary analysis were also consistent with those in the primary analysis for both outcomes (Fig 3 and S1 Appendix, Table C). The unadjusted risk ratios did not materially differ from the risk ratios adjusted for maternal characteristics (Table 2 and S1 Appendix, Table C).

**Table 2:** Perinatal outcomes after induction of labour compared with expectant management (primary analysis).

Outcome	Week of gestation induction was performed	Induction group	Expectant management group (delivery beyond week of induction)	Univariate analysis		Multivariable analysis	
		*n* (%)	*n* (%)	RR	95% CI	adjRR	95% CI
In-hospital perinatal death	39	3 (0.08)	123 (0.22)	0.36	(0.12 to 1.15)	0.37	(0.12 to 1.15)
	40	5 (0.08)	74 (0.26)	0.32	(0.13 to 0.80)*	0.33	(0.13 to 0.80)*
	41	5 (0.07)	19 (0.30)	0.25	(0.10 to 0.62)**	0.24	(0.09 to 0.65)**
Stillbirth	39	2 (0.05)	99 (0.18)	0.30	(0.07 to 1.23)	0.31	(0.08 to 1.26)
	40	3 (0.05)	61 (0.22)	0.23	(0.07 to 0.75)*	0.25	(0.08 to 0.79)*
	41	3 (0.04)	15 (0.24)	0.18	(0.06 to 0.58)**	0.18	(0.05 to 0.65)**
Birth injury	39	15 (0.40)	249 (0.45)	0.90	(0.54 to 1.52)	0.87	(0.52 to 1.46)
	40	28 (0.47)	137 (0.49)	0.98	(0.65 to 1.46)	0.96	(0.63 to 1.46)
	41	24 (0.33)	41 (0.65)	0.63	(0.41 to 0.97)*	0.47	(0.29 to 0.78)**
Shoulder dystocia	39	42 (1.13)	602 (1.08)	1.05	(0.77 to 1.43)	0.87	(0.64 to 1.19)
	40	66 (1.12)	308 (1.09)	1.02	(0.78 to 1.33)	0.85	(0.65 to 1.11)
	41	64 (0.88)	75 (1.20)	0.76	(0.58 to 1.00)	0.68	(0.49 to 0.94)*
Hypoxia in labour^a^	39	219 (5.90)	4,310 (7.73)	0.76	(0.67 to 0.87)***	0.74	(0.65 to 0.85)***
	40	492 (8.33)	2,367 (8.40)	0.99	(0.90 to 1.09)	0.98	(0.89 to 1.08)
	41	645 (8.89)	560 (8.92)	1.08	(0.99 to 1.17)	1.02	(0.91 to 1.13)
Meconium aspiration syndrome	39	6 (0.16)	414 (0.74)	0.22	(0.10 to 0.49)***	0.22	(0.10 to 0.49)***
	40	26 (0.44)	242 (0.86)	0.51	(0.34 to 0.77)**	0.52	(0.35 to 0.78)**
	41	41 (0.57)	62 (0.99)	0.67	(0.48 to 0.93)*	0.57	(0.39 to 0.83)**
Seizures^a^	39	12 (0.32)	143 (0.26)	1.26	(0.70 to 2.27)	1.14	(0.62 to 2.10)
	40	12 (0.20)	78 (0.28)	0.73	(0.40 to 1.35)	0.67	(0.36 to 1.24)
	41	15 (0.21)	23 (0.37)	0.74	(0.43 to 1.29)	0.50	(0.26 to 0.99)*
Neonatal readmission within 28 days of birth^a^	39	119 (3.20)	1,534 (2.75)	1.16	(0.97 to 1.30)	1.16	(0.96 to 1.30)
	40	192 (3.25)	709 (2.52)	1.29	(1.04 to 1.50)**	1.30	(1.03 to 1.50)**
	41	176 (2.43)	160 (2.55)	0.90	(0.76 to 1.00)	0.95	(0.76 to 1.10)
Emergency caesarean section^a^^,^^b^^,^^c^	39	1,301 (35.02)	15,992 (28.67)	1.22	(1.17 to 1.28)***	1.04	(0.99 to 1.09)
	40	2,312 (38.94)	9,409 (33.38)	1.17	(1.13 to 1.22)***	1.05	(1.01 to 1.09)*
	41	2,994 (41.27)	2,636 (42.00)	1.26	(1.23 to 1.31)***	0.94	(0.90 to 0.97)**
Instrumental delivery^a^^,^^b^^,^^c^	39	994 (26.76)	15,414 (27.63)	0.97	(0.92 to 1.02)	1.04	(0.98 to 1.10)
	40	1,647 (27.88)	7,894 (28.00)	1.00	(0.95 to 1.04)	1.06	(1.01 to 1.11)*
	41	2,024 (27.90)	1,699 (27.07)	1.00	(0.96 to 1.04)	1.06	(1.00 to 1.12)
3rd or 4th degree tears^a^	39	121 (3.26)	1,945 (3.49)	0.93	(0.78 to 1.12)	0.97	(0.81 to 1.17)
	40	183 (3.10)	973 (3.45)	0.90	(0.77 to 1.05)	0.93	(0.79 to 1.10)
	41	216 (2.98)	208 (3.31)	0.85	(0.74 to 0.98)	0.91	(0.75 to 1.10)
Maternal readmission within 28 days of giving birth^a^	39	114 (3.07)	1,120 (2.01)	1.52	(1.26 to 1.80)***	1.38	(1.13 to 1.60)**
	40	146 (2.47)	543 (1.93)	1.28	(1.07 to 1.50)**	1.16	(0.96 to 1.30)
	41	156 (2.15)	118 (1.88)	1.08	(0.91 to 1.00)	1.06	(0.83 to 1.30)

*P < 0.05

** *P* < 0.01

*** *P* < 0.001. Estimates were adjusted for pregnancy-related conditions when these were found to have significant coefficient: ^a^preeclampsia/pregnancy-induced hypertension/edema in pregnancy

^b^gestational diabetes

^c^abnormal fluid volume (oligohydramnios or polyhydramnios).

Abbreviations: adjRR, adjusted risk ratio; RR, risk ratio (unadjusted).

The NNT analysis indicated that 562 (95% CI 366–1,210) and 658 (95% CI 421–1,506) inductions of labour at 40 weeks would be required to prevent 1 perinatal death, for the primary and secondary analysis, respectively.

### Perinatal morbidity

In the primary analysis, labour induction from 39 weeks onwards was associated with a significantly reduced rate of meconium aspiration syndrome, when compared with expectant management (Fig 3). Induction at 39 weeks was also significantly associated with reduced rates of hypoxia in labour (adjRR 0.74; 95% CI 0.65–0.85). However, this association was not significant at later weeks of gestation. Labour induction at 40 weeks was associated with higher rates of neonatal readmission to hospital within 28 days of birth (adjRR 1.30; 95% CI 1.03–1.50). Induction at 41 weeks was associated with reduced rates of birth injury (adjRR 0.47; 95% CI 0.29–0.78) and neonatal seizures (adjRR 0.50; 95% CI 0.26–0.99). No differences were found in the rates of shoulder dystocia in association with induction. Similar observations were seen in the secondary analysis, although the association with neonatal seizures was not replicated in secondary analysis (adjRR 0.67, 95% CI 0.38–1.16), and the association with higher neonatal readmission was seen at 39 as well as 40 weeks (Fig 3 and S1 Appendix, Table C).

### Maternal outcomes

In the primary analysis, no differences in the rates of emergency caesarean section or instrumental delivery were found in association with induction at 39 weeks when compared with expectant management (Fig 3). Induction at 40 weeks was associated with a slightly increased risk of instrumental vaginal delivery (adjRR 1.06; 95% CI 1.01–1.11) and emergency caesarean delivery (adjRR 1.05; 95% CI 1.01–1.09). Induction at 41 weeks was associated with a slightly reduced risk of emergency caesarean section (adjRR 0.94; 95% CI 0.90–0.97) compared with expectant management. No differences were found in the rates of severe perineal tears in association with induction. Induction at 39 weeks was associated with higher risk of maternal readmission within 28 days of delivery (adjRR 1.38; 95% CI 1.13–1.60). In the secondary analysis, induction from 39 weeks onwards was associated with a 20%–30% increased rate of emergency caesarean section when compared with expectant management and a 10% increased rate of instrumental delivery at 39 and 40 weeks (S1 Appendix, Table C).

## Discussion

The key finding of the present study is that induction of labour at 40 weeks of gestation was associated with a third of the risk of perinatal death compared with expectant management in a national cohort of nulliparous women aged ≥35 years. At this stage in pregnancy, the risk of perinatal death with expectant management was 26 per 10,000 pregnancies, whereas the risk among women induced at 40 weeks was 8 per 10,000 pregnancies. If these associations are causal, these data indicate that 562 (95% CI 366–1,210) inductions of labour would be required to prevent each perinatal death. Induction of labour was also associated with a significantly reduced risk of meconium aspiration syndrome.

A recent RCT has demonstrated no short-term adverse effect on the mother or infant of routine induction of labour at 39 weeks for women of advanced maternal age, and an associated economic evaluation has suggested that such a policy was associated with lower healthcare costs, principally through reduced rates of maternal readmission [24]. These findings could be taken together with those of the present study and used to support a policy of actively offering all women aged ≥35 years in their first pregnancy to have labour induced around their due date.

However, it could be argued that, because the data in this study are observational, the findings may be due to bias. We do not feel that this is a plausible explanation. First, the women being induced had higher proportions of risk factors, such as very advanced age and acquired complications of pregnancy. Second, we were able to adjust for these and other confounding factors in our statistical models, and this was without material effect. Third, while the risk adjustment models did not include all potential confounders, if there was an imbalance in their distribution across the 2 groups, it is likely that the confounders would be more prevalent in the induction group. Fourth, other well-conducted observational studies using other datasets have found similar effects on perinatal mortality, albeit not in this specific maternal age group [12].

The major cause of perinatal death at term is antepartum stillbirth. It is biologically plausible that stopping pregnancy at week 40 prevents the possibility of an antepartum stillbirth at 41 weeks. It may be argued that induction of labour in this context should only be widely recommended when these results are confirmed by an RCT, but the rarity of the outcome means a trial would be difficult. A sample size calculation based on the observed rates of perinatal death and effect size in the present study indicates that around 15,000 women would be required for a trial with 90% power to detect similarly large effects at 40 weeks of gestation. A well-funded multicentre RCT managed to recruit just over 600 such women [11], which is 4% of the required sample size.

In our primary analysis, we observed a 5% increase in the rate of emergency caesarean section and a 6% increase in the rate of operative vaginal delivery in the induction group. Although the 35/39 trial demonstrated no statistically significant association between induction and these outcomes, the point estimates for the effects in the present study are within the 95% CIs reported in the RCT [11]. Given the higher-risk nature of the women being induced, the adverse associations with induction of labour may be due to unmeasured confounders, as discussed above. These issues may be resolved by a larger-scale RCT of routine induction of labour in 6,000 nulliparous women aged ≥35 years, which is currently in progress [25]. However, that study will not be powered to detect a reduction in the risk of perinatal death, on the basis of the sample size calculation above.

The present study had a number of methodological strengths. The cohort is large and was drawn from an unselected population-based database which records the necessary data items for the appropriate comparison groups to be defined for this study. The use of a novel technique to link mothers’ and babies’ hospital records [19] enabled an examination of both maternal and perinatal outcomes, including morbidity as well as mortality. We were also able to follow up newborns after hospital discharge to examine emergency hospital readmission rates. A disadvantage of hospital administrative birth data is varying data quality between hospitals, which led to the records from some hospitals being excluded from this study. However, this approach has been demonstrated by others to be a valid way of constructing birth cohorts [16], and validation studies have found that the data are nationally representative for key variables, including sex, gestation, birth weight, maternal age, stillbirth, and multiple birth [19].

Despite the large sample size in the present study, it could be argued that because the number of observed perinatal deaths in the induced group is very small, under-recording of labour induction in the dataset could have a major impact on the results. However, since HES is used to guide the reimbursement of maternity care expenses and labour induction is recognised within the national pricing framework [26], we would expect hospitals not to overlook this procedure when coding. To reduce the risks associated with under- and over-coding of induction, we also excluded hospitals that appeared to have divergent coding practices from the analysis (S1 Appendix, Table A).

As with other studies using routine data to examine this issue, we addressed the limitation of gestational age being recorded in weeks rather than in days by testing the robustness of our primary definition of the expectant management group using a secondary analysis that used the alternative definition [12]. Nevertheless, we were not able to control for some important possible confounders, such as maternal obesity, nor to examine some important outcomes, such as postpartum haemorrhage. It is also possible that a small number of antepartum stillbirths were inappropriately included in the expectant management group in the case of delay between death and delivery following induction. Inclusion of these women in the expectant management group would overestimate the risk of perinatal death rate in this group. However, this bias is more likely to affect the results of our secondary analysis, and we found that the magnitude of effect was similar in both analyses.

In summary, our results suggest that among women aged ≥35 years, induction of labour at term is associated with a lower rate of perinatal mortality and morbidity. Hence, bringing forward the routine offer of induction of labour from the current recommendation of 41–42 weeks to 40 weeks of gestation in this group may reduce overall rates of perinatal death. It is, however, important to note the potential for downsides to a policy which would significantly increase the use of labour induction, and further studies should examine the impact of such a policy on resource utilisation and patient satisfaction.

## Supporting information

S1 STROBE Checklist (DOCX)

S1 Appendix Includes Text A. Hospital-level data quality assessments; Text B. Analysis history; Table A. Definition; Table B. Comparison of included and excluded deliveries; and Table C. Perinatal outcomes after induction of labour compared with expectant management (secondary analysis).
(DOCX)

## Abbreviations

adjRR: adjusted risk ratio
HES: Hospital Episode Statistics
NHS: National Health Service
NNT: number needed to treat
RCT: randomised controlled trial
