# Supplementary material for: Perinatal mortality associated with induction of labour versus expectant management in nulliparous women aged 35 years or over: An English national cohort study
Source: PLoS Med. 2017 Nov 14;14(11):e1002425. doi: 10.1371/journal.pmed.1002425 (PMC5685438; doi:10.1371/journal.pmed.1002425)
Supplement: S1 Appendix — Includes Text A. Hospital-level data quality assessments; Text B. Analysis history; Table A. Definition; Table B. Comparison of included and excluded deliveries; and Table C. Perinatal outcomes after induction of labour compared with expectant management (secondary analysis). (DOCX) [file pmed.1002425.s002.docx]

**S1 Appendix.**

Hannah E Knight, David A Cromwell, Ipek Gurol-Urganci, Katie Harron, Jan H van der Meulen, Gordon CS Smith.

**Contents**

**P2 Text A. Hospital-level data quality assessments**

**P3 Table A. Definitions**

**P4 Table B. Comparison of included and excluded deliveries**

**P5 Table C. Perinatal outcomes after induction of labor compared with expectant management (secondary analysis)**

**P7 Text B. Analysis history**

**Text A. Hospital-level data quality assessments**

Hospitals were excluded from the analysis if they failed one or more of the following assessments:

**Birth status**

- More than 30% of birth records were missing the birth status field

and/or

- The total stillbirth rate was less than 1 per 1,000 or more the 10 per 1,000. These cut-offs were set based on the trust-level stillbirth rates reported in the 2014 MBRRACE perinatal mortality report.^1^

and/or

- More than 20% of stillbirths had ‘unknown timing’

**Onset of labor and delivery**

- More than 30% of birth records were missing the onset of labor field

and/or

- The total induction of labor rate was less than 10% or more than 40%

**Gestational age**

- More than 30% of birth records were missing the gestational age field

and/or

- The proportion of all births that took place between 39 and 42 completed weeks of gestation was less than 60% or more than 90**%**

^1^ Manktelow BN, Smith LK, Seaton SE, et al. MBRRACE-UK Perinatal Mortality Surveillance Report, UK Perinatal Deaths for Births from January to December 2014. Leicester: The Infant Mortality and Morbidity Studies, Department of Health Sciences, University of Leicester; 2016.

**Table A. Definitions**

| **Category** | **Variable** | **Codes used** |
| --- | --- | --- |
| **Induction** | **Delivery onset** | Delivery onset (delonset) 3-5 |
| **Co-morbidities** | **Gestational diabetes** | ICD-10: O24.X |
|  | **Fluid abnormality** | ICD-10: O40.X; O41.1 |
|  | **Pre-eclampsia** | ICD-10: O12.X-O16.X |
| **Outcomes** | **Emergency (intrapartum) cesarean section** | OPCS: R18.X; R25.1. Birth episodes with delivery onset (delonset) coded as 2 ‘no labour – caesarean section’ were classified as prelabour caesarean sections. |
|  | **Instrumental delivery** | OPCS: R21.X-R22.X |
|  | **3^rd^/4^th^ degree perineal tear** | ICD-10: O70.2-3 |
|  | **Maternal/neonatal readmission** | Babies/mothers readmitted with the following admission method codes: 21, 22, 23, 24, 28, 2A, 2B, 2D, 31, 32, 82, 83 within 28 days of the birth episode. The discharge date from the readmission must be at least one day after the readmission date. Planned transfers are identified as follows: the admission date of the second spell is within plus/minus one day of the discharge date. Either the first spell has a discharge destination of 51 or 52, or the second spell has an admission source of 51 or 52, or the second spell has an admission method of 81. Deaths within the delivery spell are identified using discharge method 4 (Died) or 5 (Baby was stillborn) and excluded from the denominator |
|  | **Stillbirth** | Value suggesting stillborn in birth status (2, 3, 4) or discharge fields (5), |
|  | **In-hospital perinatal death** | Value suggesting stillborn in birth status (2, 3, 4) or discharge fields (5), or discharged within 7 days with discharge method (4) or discharge destination (79) suggesting death, or attended A&E within 7 days of birth and was either brought in dead (70) or died in the department (10) |
|  | **Birth injury** | ICD-10: P10.X-11.X; P13.X-14.X |
|  | **Shoulder dystocia** | ICD-10: O66.0; P03.1; S14.3; P14.X |
|  | **Hypoxia in labor** | ICD-10: P20.1 |
|  | **Seizures** | ICD-10: P90.X; R56.X |
|  | **Meconium aspiration** | ICD-10: P24.0 |
| **Exclusion criteria** | **Prelabour caesarean section** | OPCS: R19 or delivery onset (delonset) 2 (no labour – caesarean section) |
|  | **PROM** | ICD-10: O42.X |
|  | **Placenta previa** | ICD-10: O44.X-45.X |
|  | **Breech/malposition** | ICD-10: O32.1; O64.1; O32.0; O32.2; O80.1; ICD-10: O83.0-1; O83.3; O36.7; O64.0; O64.2-5; O64.8-9  OPCS: R19.X-20.X  Delmeth: 5-6 |
|  | **Cardiac condition** | ICD-10: I00.X-I02.X; I05.X-I09.X; I2.X; I31.X; I4.X; I51.X-52.X; I6.X-7.X |
|  | **Pre-existing hypertension** | ICD-10: O10.X-O11.X; I1.X |
|  | **Pulmonary condition** | ICD-10: JXX.X (except J45.X); I26.X-I28.X |

**Table B. Comparison of included and excluded deliveries**

| **Characteristic** | **Group** | **Eligible women in hospitals with good quality data**  **n (%)** | **Eligible women in hospitals with bad quality data** |
| --- | --- | --- | --- |
| **Maternal age** | Age 35-39 | 63,571 (82.2) | 41,197 (81.2) |
|  | Age 40-50 | 13,756 (17.8) | 9,564 (18.8) |
| **Maternal ethnicity** | White ethnicity | 56,848 (81.0) | 35,390 (79.8) |
|  | Asian ethnicity | 4,805 (6.8) | 3,433 (7.7) |
|  | Black ethnicity | 3,879 (5.5) | 2,741 (6.2) |
|  | Other ethnicity | 4,676 (6.7) | 2,803 (6.3) |
| **Maternal socioeconomic status quintile** | SES 1 (least deprived) | 16,915 (21.9) | 12,006 (23.7) |
|  | SES 2 | 16,305 (21.1) | 10,811 (21.3) |
|  | SES 3 | 15,660 (20.3) | 10,882 (21.4) |
|  | SES 4 | 16,717 (21.6) | 9,659 (19.0) |
|  | SES 5 (most deprived) | 11,726 (15.2) | 7,403 (14.6) |
| **Year of birth** | 2009 | 17,044 (22.0) | 10,336 (20.4) |
|  | 2010 | 15,733 (20.4) | 10,811 (21.3) |
|  | 2011 | 16,104 (20.8) | 10,444 (20.6) |
|  | 2012 | 14,738 (19.1) | 10,047 (19.8) |
|  | 2013 | 13,708 (17.8) | 9,123 (18.0) |
| **Birthweight centile** | BW 10-90th centile | 64,702 (83.7) | 32,840 (64.7) |
|  | BW <10th centile | 7,142 (9.2) | 3,849 (7.6) |
|  | BW >90th centile | 5,483 (7.1) | 3,123 (6.2) |
| **Sex of baby** | Male sex | 39,556 (51.2) | 25,984 (51.2) |
| **Pregnancy complications** | Preeclampsia | 5,812 (7.5) | 4,165 (8.2) |
|  | Gestational diabetes | 2,315 (3.0) | 2,379 (4.7) |
|  | Abnormal fluid volume | 657 (0.9) | 553 (1.1) |

**Percentage of records missing key data items, according to results of data quality assessments**

| **Data item** | **Good quality hospitals (%)** | **Bad quality hospitals (%)** |
| --- | --- | --- |
| **Gestational age** | 10.8 | 40.3 |
| **Method of onset of labour** | 10.5 | 24.1 |
| **Birth status** | 9.8 | 25.2 |

**Table C. Perinatal outcomes after induction of labor compared with expectant management (secondary analysis)**

| **Neonatal Outcome** | **Week of gestation induction was performed** | **Induction group** | **Expectant management group (delivery at or beyond week of induction)** | **Univariate analysis** | | **Multivariable analysis** | |
| --- | --- | --- | --- | --- | --- | --- | --- |
|  |  | **n (%)** | **n (%)** | **RR** | **95% CI** | **RR** | **95% CI** |
| In-hospital perinatal death | 39 | 3 (0.08) | 159 (0.22) | 0.37 | (0.12 to 1.17) | 0.38 | (0.12 to 1.17) |
|  | 40 | 5 (0.08) | 118 (0.24) | 0.36 | (0.15 to 0.88)* | 0.36 | (0.15 to 0.87)* |
|  | 41 | 5 (0.07) | 69 (0.33) | 0.21 | (0.08 to 0.52)** | 0.22 | (0.09 to 0.54)** |
| Stillbirth | 39 | 2 (0.05) | 128 (0.11) | 0.31 | (0.08 to 1.25) | 0.31 | (0.08 to 1.25) |
|  | 40 | 3 (0.05) | 96 (0.19) | 0.26 | (0.08 to 0.83)* | 0.27 | (0.08 to 0.84)* |
|  | 41 | 3 (0.04) | 58 (0.28) | 0.15 | (0.05 to 0.48)** | 0.16 | (0.05 to 0.50)** |
| Birth injury | 39 | 15 (0.40) | 302 (0.41) | 0.98 | (0.59 to 1.65) | 0.95 | (0.57 to 1.59) |
|  | 40 | 28 (0.47) | 221 (0.44) | 1.07 | (0.72 to 1.58) | 1.06 | (0.71 to 1.58) |
|  | 41 | 24 (0.33) | 113 (0.54) | 0.61 | (0.39 to 0.95)* | 0.60 | (0.39 to 0.93)* |
| Shoulder dystocia | 39 | 42 (1.13) | 732 (0.99) | 1.14 | (0.83 to 1.55) | 0.99 | (0.73 to 1.36) |
|  | 40 | 66 (1.12) | 536 (1.07) | 1.04 | (0.81 to 1.34) | 0.89 | (0.68 to 1.15) |
|  | 41 | 64 (0.88) | 244 (1.17) | 0.76 | (0.58 to 1.00)* | 0.70 | (0.53 to 0.92)* |
| Hypoxia in labor ^a^ | 39 | 219 (5.90) | 5,062 (6.88) | 0.86 | (0.75 to 0.98)* | 0.83 | (0.73 to 0.94)** |
|  | 40 | 492 (8.33) | 3,818 (7.65) | 1.09 | (0.99 to 1.19) | 1.07 | (0.97 to 1.17) |
|  | 41 | 645 (8.89) | 1,722 (8.23) | 1.08 | (0.99 to 1.18) | 1.09 | (1.00 to 1.19) |
| Meconium aspiration | 39 | 6 (0.16) | 471 (0.64) | 0.25 | (0.11 to 0.56)** | 0.26 | (0.11 to 0.57)** |
|  | 40 | 26 (0.44) | 388 (0.78) | 0.57 | (0.38 to 0.84) | 0.56 | (0.37 to 0.83)** |
|  | 41 | 41 (0.57) | 201 (0.96) | 0.59 | (0.42 to 0.82)** | 0.59 | (0.42 to 0.82)** |
| Seizures ^a^ | 39 | 12 (0.32) | 172 (0.23) | 1.38 | (0.77 to 2.48) | 1.21 | (0.66 to 2.22) |
|  | 40 | 12 (0.20) | 131 (0.26) | 0.77 | (0.43 to 1.40) | 0.70 | (0.38 to 1.26) |
|  | 41 | 15 (0.21) | 63 (0.30) | 0.69 | (0.39 to 1.21) | 0.67 | (0.38 to 1.16) |
| Neonatal readmission within 28 days of birth ^a^ | 39 | 119 (3.20) | 1,534 (2.08) | 1.54 | (1.28 to 1.85) *** | 1.54 | (1.28 to 1.86)*** |
|  | 40 | 192 (3.25) | 1,342 (2.69) | 1.21 | (1.04 to 1.40) * | 1.20 | (1.03 to 1.40)* |
|  | 41 | 176 (2.43) | 533 (2.55) | 0.95 | (0.81 to 1.13) | 0.95 | (0.80 to 1.12) |
| Emergency cesarean section ^a,b,c^ | 39 | 1,391 (35.02) | 18,527 (25.17) | 1.39 | (1.33 to 1.46)*** | 1.19 | (1.14 to 1.25)*** |
|  | 40 | 2,312 (39.13) | 13,680 (27.43) | 1.43 | (1.38 to 1.48)*** | 1.26 | (1.22 to 1.31)*** |
|  | 41 | 2,994 (41.27) | 6,415 (30.64) | 1.35 | (1.30 to 1.39)*** | 1.28 | (1.24 to 1.33)*** |
| Instrumental delivery ^a,b,c^ | 39 | 994 (26.76) | 18,811 (25.55) | 1.05 | (0.99 to 1.11) | 1.12 | (1.06 to 1.19)** |
|  | 40 | 1,647 (27.88) | 13,767 (27.60) | 1.01 | (0.97 to 1.05) | 1.08 | (1.03 to 1.13)*** |
|  | 41 | 2,024 (27.90) | 5,870 (28.04) | 1.00 | (0.95 to 1.04) | 1.02 | (0.97 to 1.06) |
| 3rd/4th degree tears ^a^ | 39 | 121 (3.26) | 2,439 (3.31) | 0.98 | (0.82 to 1.18) | 1.04 | (0.87 to 1.24) |
|  | 40 | 183 (3.10) | 1,762 (3.553) | 0.88 | (0.75 to 1.02) | 0.94 | (0.80 to 1.09) |
|  | 41 | 216 (2.98) | 757 (3.62) | 0.82 | (0.71 to 0.96)* | 0.83 | (0.71 to 0.96)* |
| Maternal readmission within 28 days of giving birth ^a^ | 39 | 114 (3.07) | 1,120 (1.52) | 2.02 | (1.67 to 2.44)*** | 1.82 | (1.49 to 2.23)*** |
|  | 40 | 146 (2.47) | 974 (1.95) | 1.27 | (1.07 to 1.50)** | 1.15 | (0.96 to 1.37) |
|  | 41 | 156 (2.15)) | 387 (1.85)) | 1.16 | (0.97 to 1.40) | 1.11 | (0.92 to 1.34) |

*p<0.05 ** p<0.01 *** p<0.001. Estimates were adjusted for pregnancy-related conditions when these were found to have significant coefficients. These are labelled in the table: a) pre-eclampsia/pregnancy-induced hypertension/edema in pregnancy; b) gestational diabetes; c) abnormal fluid volume (oligohydramnios or polyhydramnios).

**Text B. Analysis history for the observational study described in: Perinatal mortality associated with induction of labour versus expectant management in nulliparous women aged 35 years or over: a national cohort study. Knight HE, Cromwell DA, Gurol-Urganci I, Harron K, van der Meulen JH, Smith GCS. PLOS Medicine.**

We did not publish or pre-register a protocol for this secondary analysis of data from Hospital Episode Statistics (HES). We followed a clear analysis plan, as described in the methods section. Further details on the analysis history are described below:

1. The study was motivated by the question “does induction of labour at >=39 weeks reduce the risk of perinatal mortality among nulliparous women aged ≥35 compared with expectant management?” which addressed some of the limitations of previously published studies on this topic.
2. The inclusion/exclusion criteria for the study were established at the outset of the study. As described in the manuscript, we extracted the records of nulliparous women aged between 35 and 50 years who had a singleton birth from the database, excluding deliveries before 39+0 weeks of gestation. We also excluded women who had a caesarean section prior to labour. We originally intended to include women with pre-existing comorbidities in the cohort and to adjust for these potential confounders in the analysis. However, once the cohort was constructed the number of women in it with existing comorbidities (type 1 diabetes, hypertensive disorders or cardiac, renal or lung disease) was very small (less than 500). Upon discussion we therefore decided to exclude these women form the cohort as national guidance recommends they are delivered before 39 weeks of gestation. This modification was made prior to the commencement of any statistical analyses
3. The statistical approach was determined at the outset of the study. The only revision made was to switch to using Poisson regression rather than logistic regression as originally planned because our analysis contains several common outcomes (see Knol et al., 2012). This modification was made prior to the commencement of any statistical analyses. As described in the manuscript, we conducted sensitivity analyses using an alternative definition of expectant management first used by Stock et al. (2012) to interrogate the robustness of our findings.
4. We did not make any changes to the analysis following feedback from the PLOS Medicine editors or reviewers, with the exception of examining and reporting the particular methods of induction of labour (medical; surgical and combined) used for the women in our cohort.
